# General Medicine and Therapeutics

**Published:** 1896-08

**Authors:** 


### GENERAL MEDICINE AND THERAPEUTICS.

### SLEEPLESSNESS.

It is unfortunate that the physiology of sleep is not better understood. It is even more unfortunate that what we do know about the phenomenon of sleep is not more diffused among the profession. How frequently indeed do we observe physicians who mistake unconsciousness for sleep, and who seemingly regard the goal of the application of their therapeutical measures as having been reached, when they have succeeded in rendering a tired, worn-out, nervous individual oblivious to his surroundings by placing him in a condition which they call sleep, but which is simply unconsciousness. An experience of several years in insane hospital practice presented to the writer, in a very strong light, the enormity of this error and its wide prevalence. It was no uncommon sight to see patients brought to the hospital in an unconscious state, due to the administration of narcotics; opium or morphine being the favorite drug used. One case is worthy of mention here, a male, neurasthenic, harmless, but suffering from the exaggerated mental symptoms, so marked in this disease, was accompanied by his physician to the hospital. The physician stated his reason for coming was, that he considered it important to keep his patient asleep, so that he would not appreciate the humiliation of entering the hospital, and further, that inasmuch as he had not been sleeping well for some time, and that he was now sleeping, he considered the opportunity a good one to let him recuperate. To this end, the physician was at intervals giving hypodermic injections of morphine, and the poor, worn-out, narcotized patient, saturated with morphine, and believed to be asleep, was, as best he could, with his worn-out nervous mechanism, fighting for his life, which his respiration, weak pulse, and inhibited reflexes showed was at very low ebb. This case was not an exceptional one; it was, alas, too common an experience. The physician had lost sight of the very essential principle which should govern our therapy in the treatment of sleeplessness, and that is, that sleep has for its object the repair of the wear and tear of vital processes of life, and to insure sleep we must not interfere with these processes, which we do when drugs are given until sedation results.

There is another thing to bear in mind in the consideration of sleeplessness, and that is, that there is a source of irritation somewhere in the economy, which, if relieved, will be followed by sleep. Again, "an axiom" well worth remembering is, that the more gentle the means employed to induce sleep, the more natural will be the sleep induced, and the more gentle the means employed, the more careful must we be to select the right time for their use. We believe that Dujardin-Beaumetz was right when speaking of the use of drugs in the treatment of sleeplessness; he said: "That for a drug to be hypnotic, it must imitate the natural condition of sleep, by effecting a lowered intracranial pressure, and that drugs which, though bringing about unconsciousness do not lower cerebral pressure, or which increase it, cannot claim to be hypnotics. Opium and morphine are objectionable on this ground. Drug treatment must put the patient in a position to go to sleep in a natural way, and not *put* him to sleep." Sedatives do this, but narcotics do not.

In the use of sedatives we must be cautious and not use them *ad libitum*. The writer, in a paper on "Sedatives in the Treatment of Insanity" (1892, *Hospital Bulletin of Minnesota*), said: "Each case is a 'law unto itself,' and as such requires patient and persistent study ere we commit the folly of giving a hypnotic, when more simple and efficacious methods would produce satisfactory results." You cannot cure sleeplessness by drug treatment; the drugs simply conserve nervous energy and act as valuable assistants to the building-up process, necessary to cure the sleeplessness. Sedatives act, as before stated, by placing the patient in a position to go to sleep, and nature does the rest.

In our experience we have learned to rely upon the bromides, chloral, cannabis indica, and hyoscyamus as sedatives, which, if judiciously used, bring order out of chaos. The bromides lower the sensibility of the brain, and thus promote sleep. The single salts can be used, but in the writer's experience, where a sedative is indicated in sleeplessness, it is better to combine them, and when there is any excitement, add chloral. Cannabis indica is a sedative which is but little used by the general practitioner, and for the reason that it is misunderstood, misrepresented, and as a result never used as it should be. Clouston, Mathison, and Echeverria have taught us their value. Hyoscyamus is another sedative, the value of which is not appreciated, a drug which is indorsed by Budde, Brush, Krafft-Ebing as a hypnotic. Now, these valuable sedatives, when combined, give us a thoroughly reliable and satisfactory agent with which to treat sleeplessness, and in the writer's experience no more elegant or reliable preparation is before the profession than that of bromidia, in which is combined in proper proportion, the bromide of potassium, chloral hydrate, hyoscyamus and cannabis indica. We feel that the profession can always rely upon this combination, and find it especially useful in the treatment of sleeplessness.—*The Medical Fortnightly.*

---

### GUAIACUM IN GOUT AND RHEUMATISM.

At the Royal Medical and Chirurgical Society, on May 26th, Sir Alfred Garrod, after speaking of numerous observations carried on for very many years among both hospital and private patients with guaiacum, both in the form of a powder and as ammoniated tincture of guaiacum, thought he had been successful in establishing the following points in regard to the action of guaiacum: (1) Guaiacum is innocuous, and may be taken for an indefinite period of time, and looked upon as a condiment rather than a drug —as harmless as ginger or any other condiment. (2) Guaiacum possesses a considerable power (but less than colchicum) in directly relieving patients suffering from gouty inflammation of any part; it may be given whenever there is but little fever. (3) Guaiacum taken in the intervals of gouty attacks has a considerable power of averting their recurrence; in fact, it is a very powerful prophylactic. (4) Guaiacum does not appear to lose its prophylactic power by long-continued use. (5) There are a few persons who cannot readily continue the use of guaiacum; for such cases there are other drugs whose action as prophylactics is in some repects similar; perhaps serpentary is one of the most powerful of these. The author gave a short account of serpentary, and mentioned the fact that he had given it successfully in gouty inflammation in the elderly subject; as a prophylactic he had less personal experience, but he did not doubt that it was possessed of considerable power. Before attempting to explain the mode of action of guaiacum in the treatment of gout, he gave his view of the origin of uric acid in the animal economy; instead of supposing that it was formed in the system by the metabolism of the nitrogenized tissues and then thrown out by the kidneys, he was of opinion that it was produced from urea and other nitrogenized bodies in the blood by the direct action of the kidneys, and that when uric acid was contained in the blood, this arose from the absorption from the kidney structures of the urate of ammonium, depending on the want of sufficient throwing-off powers from these tissues. The author was aware that it was from his own discovery of uric acid in the blood that the opposite view of the formation of uric acid arose. To explain the prophylactic action of guaiacum the author did not think that it affected the formation of uric acid, but that it acted directly on the kidney itself as a stimulant, and enabled it to get rid of any accumulation in the tubules, and thus prevent absorption from them into the blood. In confirmation of this view it was found that patients, when taking guaiacum, often had unusual deposits of urates in their urine. The paper closed with the following remarks: "If the statements contained in this communication have the effect of inducing my medical brethren to direct their attention to the value of guaiacum in the treatment and prevention of gout, I feel I shall have conferred some benefit on the profession and through them on the public at large."

Sir A. Garrod was quite certain that urate of sodium was to be found in every gouty joint. He quite agreed that guaiacum was useful in chronic rheumatism, especially in muscular affections. From an examination of the late Dr. Seymour's cases, made many years ago, he was certain that some described as acute rheumatism and relieved by guaiacum were in reality gouty. Healthy skin never allowed uric acid to pass through in sweating; he had examined as much as ten ounces of sweat, and had never found any uric acid. Uric acid could, of course, be obtained from the fluid of a blister. —*Brit. Med. Jour.*, May 30, '96.

